# Another Medical Reformer

**Published:** 1907-07-13

**Authors:** F. W. Forbes Ross

**Affiliations:** 53 Harley Street


### EDITOR'S LETTER-BOX.

Our Correspondents are reminded that prolixity is a great bar to publication, and that brevity of style and conciseness of statement greatly facilitate early insertion.]

"ANOTHER MEDICAL REFORMER."

*To the Editor of* THE HOSPITAL.

SIR,—I am obliged for the copy of your paper sent to me, specially marked for my edification. If you will read it again, you will see that it contains an abominable imputation which amounts to a gross slander and libel of me. It is a pity that your rage prevents you from seeing that you have given away your case and proved mine for me up to the hilt. For this I am grateful to you and bear you no ill will. Avoid personalities; they are vulgar.

I am yours faithfully,

F. W. FORBES ROSS, M.D., F.R.C.S.Eng.

53 Harley Street, July 1.

[We publish this letter as it affords a fair illustration of Dr. Ross's epistolary style and controversial method. And as he is so well satisfied with the substance and effect of our article, it is appropriate he should have an opportunity of recording his gratitude. Our readers will judge for themselves of his qualifications as an authority on the introduction of vulgar personalities into the discussion of a public question.—ED. THE HOSPITAL.]

THE HOSPITAL *July* 13, 1907.

